# Editorial: Optimizing psychosocial work environments and experiences for people working in isolated, confined, and/or extreme conditions

**DOI:** 10.3389/fpsyg.2025.1581336

**Published:** 2025-04-14

**Authors:** Megan Woods, Shanique G. Brown, Kimberley Norris

**Affiliations:** ^1^Tasmanian School of Business and Economics, University of Tasmania, Hobart, TAS, Australia; ^2^City University of New York, New York, NY, United States; ^3^School of Psychological Sciences, University of Tasmania, Hobart, TAS, Australia

**Keywords:** isolated, contained, extreme, psychosocial, work, team, social support, lunar

People who work in isolated, confined, and/or extreme (ICE) environments often work in conditions which are physically and psychologically hazardous and limit access to resources and support beyond those immediately and locally available. These conditions introduce additional hazards and opportunities for worker functioning, productivity, and growth into psychosocial work environments (PWEs), generally by increasing demands on workers and reducing the resources available to deal with them.

The seven articles in this Research Topic examine aspects of PWEs in Arctic, space, prisons, long-distance truck driving, fly-in-fly-out (FIFO) work and online ICE work environments. They provide new insights into PWEs in ICE work contexts by examining the psychosocial hazards—and the impacts on workers—posed by physical isolation, separation from non-work life, operation in closed work systems, loneliness, and forced proximity to colleagues who both heighten and alleviate the stressors that individuals experience. Five of the articles also advance recent work on the positive impacts of ICE environments on workers' self-efficacy, self-reliance, personal discovery (Blight and Norris, 2019) and resilience (Harrison et al., 2021) by emphasizing how workers create social support for themselves and others in such contexts. Podgorski et al. explored how demands and resources of organizational justice, job dangerousness and job stress impacted job involvement and job satisfaction of Slovenian prison officers working in a closed system work environment. Unsworth and Seivwright examined how FIFO workers navigate the necessity of managing multiple and separate identities at home and away on work sites. Bell et al. examined how long-term isolation, confinement and support source impacted the perceived social support (PSS) of crewmembers in five spaceflight analog missions. They found PSS declined over time and was more impactful when provided by other crew members, highlighting the criticality of support from peers in ICE work. Marques-Quinteiro et al. illustrated *how* this can occur by examining how members of a two-person lunar analog mission adapted their behavioral and affective responses to the evolving challenges, focus and needs of their mission to support themselves and each other. Landon et al. examined, and developed new measures for, group living skills (GLS) as an influence on the supportiveness (or stressfulness) of PWEs experienced by 24 teams in space and space analog environments. Their findings highlight specific skills that workers need, and may need to develop, to support themselves and others in contained work environments. Martinescu and Beersma demonstrated that long-distance truck drivers in Europe use gossip to reduce demands, enhance resources and social support, and enable emotion-focused coping and problem-focused coping for themselves and other truck drivers. Zhou et al. examined how online workers offset isolation and loneliness finding that the use of enterprise social media usage had a positive effect on workplace loneliness but this was moderated by ICT hassle, such as system failures.

The articles in this Research Topic offer promising directions for future research about optimizing PWEs in ICE work conditions. Increased interest in unique work environments, commercial developments in Arctic and space tourism, and increased adoption of virtual reality and AI-supported tools for social and psychological support (Anderson et al., 2023; Kim et al., 2024; Ren et al., 2024; Wiederhold, 2025) will likely provide more. For example, commercial space travel will involve longer-term confinement and crews that include non-scientists and diverse travel motivations, necessitating expansion of past research that has primarily examined crews of astronauts traveling for scientific purposes, and/or with previous experience in isolated environments. Technological advancements will enable investigation of novel support tools, such as virtual and augmented reality, for alleviating isolation, providing social support, and enhancing mental health (Holt, 2023; Thomas, 2023), and potentially enabling explorations of PWEs without real-time real-world access to ICE work sites.

Leveraging these opportunities depends, however, on researchers, reviewers and audiences understanding ICE work research also presents specific challenges and success factors evidenced in this Research Topic. These include: theorizing which accounts for the specialized contexts of ICE environments (i.e., constructivism) rather than extends generalizable theory to these settings (i.e., positivism); research and statistical methods (e.g., qualitative, experience sampling, non-frequentist approaches) which maximize the usefulness of small sample sizes (a feature of, and valuable to, this work); and the criticality of co-design with industry partners, interdisciplinary teams, insights from team science (see Kane and Emich, 2024) and the pragmatic use of analog environments to accommodate the cost, rarity and operational imperatives of ICE work sites.
